# Virome analysis of ticks in a forest region of Liaoning, China: characterization of a novel hepe-like virus sequence

**DOI:** 10.1186/s12985-021-01632-x

**Published:** 2021-08-09

**Authors:** Zijun Yang, Ju Zhang, Shixing Yang, Xiaochun Wang, Quan Shen, Guangming Sun, Hao Wang, Wen Zhang

**Affiliations:** 1 Department of Microbiology, School of Medicine, Jiangsu University, Zhenjiang, 212013 Jiangsu China; 2 Department of Clinical Laboratory, Huai’an Hospital, Xuzhou Medical University, Huai’an, 223002 Jiangsu China; 3 Xuzhou Central Hospital, 131 Huanchen Road, Xuzhou, 221009 China

**Keywords:** Virome of ticks, Metagenomic analysis, Dabieshan tick virus, Tick-borne hepe-like virus, Liaoning Province

## Abstract

**Background:**

Ticks (class *Arachnida*, subclass *Acari*) are vectors of transmitting a broad range of pathogenic microorganisms, protozoa, and viruses affecting humans and animals. Liaoning Province is rich in forests where different animals and, abundant *Haemaphysalis longicornis* ticks exist.

**Methods:**

Using viral metagenomics, we analyzed the virome in 300 *Haemaphysalis longicornis* ticks collected from June to August 2015 in the forested region of Liaoning Province, China.

**Results:**

From the 300 ticks, 1,218,388 high-quality reads were generated, of which 5643 (0.463%) reads showed significant sequence identity to known viruses. Sequence and phylogenetic analysis revealed that viral sequences showing a close relationship with Dabieshan tick virus, Aleutian mink disease virus, adeno-associated virus, Gokushovirus, avian gyrovirus 2 were present in the virome of these ticks. However, the significance of these viruses to human and animal health requires further investigation. Notably, an hepe-like virus, named tick-borne hepe-like virus sequence, was obtained and was highly prevalent in these ticks with a rate of 50%. Nevertheless, one constraint of our study was the limited geographical distribution of the sampled ticks.

**Conclusion:**

Our study offers an overview of the virome in ticks from a forest region of Liaoning Province and provides further awareness of the viral diversity of ticks.

**Supplementary Information:**

The online version contains supplementary material available at 10.1186/s12985-021-01632-x.

## Background

Ticks (class *Arachnida*, subclass *Acari*) transmit a broad range of pathogenic microorganisms, protozoa, and viruses and are the second most common vectors of diseases affecting livestock, humans, and companion animals [1, 2]. The diseases caused by the tick-borne virus are numerous and severe. In Africa, Asia, and Europe, 158 cases of CCHFV (Crimean-Congo hemorrhagic fever virus) infection were published from 1953 to 2016, with an overall case fatality rate of 32.4% [3]. SFTS (Severe fever with thrombocytopenia syndrome), with a 5.3% national average mortality rate, was reported in 23 provinces of China, with increased numbers yearly from 2010 to 2016 [4]. Moreover, the incidence of some tick-borne infections and transmissions in recent decades showed an increasing or fluctuating tendency due to various factors, mainly associated with increased tick-exposure, especially with the enlargement of cities, taking the place of forests, and exposing wild hosts to humans and livestock animals [5–7].

The rise of metagenomics analysis has transformed virus discovery and revealed a remarkable diversity of viruses sampled from ticks [8, 9]. Researchers found a large monophyletic group of emerging viruses and named this putative new virus family 'Chuviridae' [9, 10]. Jingmen tick virus, a segmented RNA virus, was detected firstly from ticks in the Jingmen region of Hubei Province in China [11]. Huaiyangshan virus, a novel species of the genus *Phlebovirus*, was found in human and tick samples with high prevalence [12, 13]. Together, these studies showed that using metagenomics can express the diversity of viruses from ticks massively.

Liaoning Province, situated in the northeastern region of China, is owned by a temperate monsoon climate region with plenty of rainfall and sunshine. Furthermore, it has 4.641 million hectares of forests and is rich in animal resources, with 827 species of animals, including amphibians, mammals, reptiles, and birds. Researchers found that tick richness correlated with forest size, even among locally common birds [14]. Ticks and tick-borne diseases have the evolution of cooperation with various wild animal hosts, and these hosts constitute reservoir hosts for ticks and tick-borne pathogens[1]. Nevertheless, with urbanization and the increasing range of human activity, a state of equilibrium between them may be off, and the risk of tick bites and infecting diseases increases. *Haemaphysalis longicornis* (*H. longicornis*) is the predominant species in Liaoning Province [15], but the overview of viruses carried by ticks has not yet been entirely elucidated. It is necessary to investigate the viruses carried by ticks and identify their natural habitats to prevent outbreaks of tick-borne viral diseases [6].

Since the necessity and feasibility, this study aims to study the diversity and evolutionary origin of viruses in ticks from a forest of Liaoning Province by a viral metagenomic approach.

## Methods

### Sample collection

**Fig. 1:**
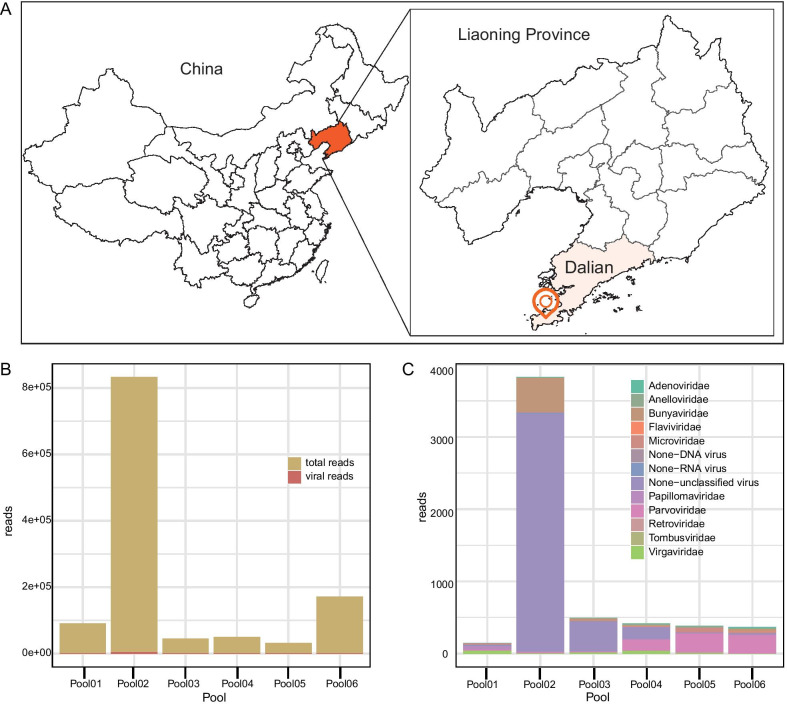
The map of collection sites and the viral reads in each pool. **A** Map of tick collection sites. **B** The proportion of viral sequences in total reads. **C** The abundance of viral reads of each family. The abundance was shown as the actual number of viral reads in each library

According to climatic conditions of summer-autumn favor tick proliferation, we collected 300 live adult ticks from June to August during 2015 in a big forest park of Dalian city in southern Liaoning (Fig. 1A) by the drag-flag method. Six different batches of fifty ticks were sampled based on sampling time (early June, late June, early July, late July, early August, and late August). Ticks were placed in labeled vials and shipped on dry ice [16]. All the collected ticks were identified using tick taxonomic keys by tick entomologists under the microscope at a magnification of × 56 [17].

### Tick sample pool preparation

The collected ticks were divided into six groups (Pool 01-06) based on their sampling month (early June, late June, early July, late July, early August, and late August) (Additional file 1). An additional table shows these pools in detail (see Additional file 1). Before homogenization, each tick pool was washed with 75% alcohol to remove contaminants on ticks and washed thrice with 1 mL of phosphate-buffered saline (PBS) to eliminate external microbes. The tick samples were homogenized, frozen, and thawed three times on dry ice, and the supernatants were then collected after centrifugation (5 min, 15,000*g*, 4 °C).

### Viral metagenomic analysis

500ul of each supernatant was filtered through a 0.45-μm filter (Millipore) to remove eukaryotic and bacterial cell-sized particles. The filtrate was treated for 60 min at 37 °C with a DNases mixture (Turbo DNase from Ambion, Baseline-ZERO from Epicentre), benzonase (Novagen), and RNase (Fermentas) to digest unprotected nucleic acid [18, 19]. Nucleic acids (total DNA and RNA) were then extracted using a QIAamp Viral RNA Mini Kit (QIAGEN) following the manufacturer's instructions. Extractions were reverse-transcribed to cDNA using reverse transcriptase (Super-Script III, Invitrogen). Total nucleic acids were subjected to RT reactions with SuperScript III reverse transcriptase (Invitrogen), following second-strand cDNA synthesis with Large (Klenow) fragment (NEB). Sixty-four libraries were then constructed using Nextera XT DNA Sample Preparation Kit (Illumina) and sequenced using the HiSeq Illumina platform with 250 base pair-ends with dual barcoding for each pool [20].

### Bioinformatics analysis

Paired-end reads of 250 bp generated by HiSeq were debarcoded using vendor software from Illumina. Clonal reads were removed, and low-sequencing-quality tails were trimmed using Phred. Adaptors were removed using the default parameters of VecScreen[21]. The cleaned reads were assembled de novo within each barcode group utilizing the ENSEMBLE assembler [22]. The assembled contigs and singlets were compared to an in-house viral proteome database using BLASTx with an E-value cutoff of < 10¯^5^. Candidate viral hits were then contrasted to an in-house non-redundant (NVNR) protein database to remove false-positive viral hits. The NVNR database was compiled using non-viral protein sequences extracted from an NCBI nr fasta file (based on annotation taxonomy, excluding the virus kingdom). Contigs without significant BLASTx similarity to the viral proteome database were searched against viral protein families in the vFam database using HMMER3 to detect remote viral protein similarities.

### Viral sequences acquisition and PCR validation

The assembled contigs and unassembled reads in known taxonomy assignments obtained from the previous step were performed de novo assembly and reference mapping in Geneious version 2019.2.3 [23] to acquire interesting viral genomes or segments. Then, nested PCR and Sanger sequencing was used to verify important fragments (i.e. TKHEV, DTV-ln, and TKGyV). Besides, genome annotation, ORF prediction and primer design were also performed using Geneious version 2019.2.3.

### Library quantification

To safeguard consistency in the library, the volume of each library for next generation sequencing (NGS) is based on the relative brightness intensity of electrophoretic bands. After the construction of the sequencing libraries, the quality of the libraries was confirmed by agarose gel electrophoresis (1% agarose). Eventually, library quantitation, including testing the concentration and fragment insertion size were performed before NGS sequencing.

### Quality control

Standard precautions were used for all steps to prevent cross-sample contamination and nucleic acid degradation. Aerosol filter pipet tips were used to reduce the possibility of sample cross-contamination. All the materials (including microcentrifuge tubes, pipet tips, and so on) directly contacted with nucleic acid samples were RNase and DNase free. Nucleic acid samples were dissolved in DEPC treated water and RNase inhibitors were added.

### Phylogenetic analysis

**Fig. 2:**
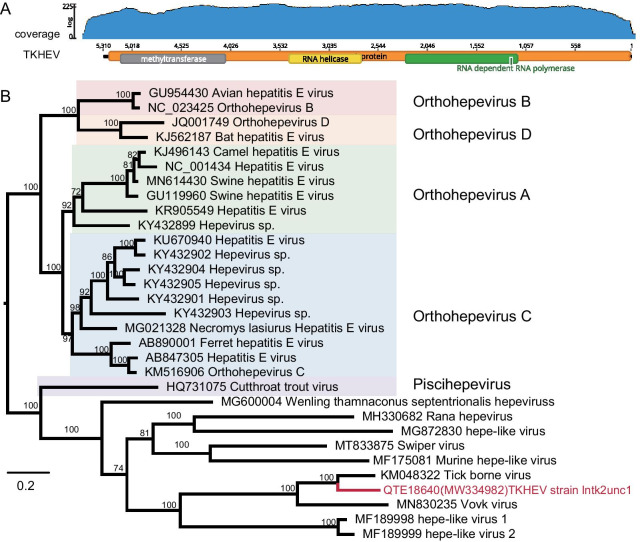
Genome structure and phylogenetic analysis of TKHEV sequence. **A** Schematic presentation of the genome of TKHEV. In the upper panel, the blue area means the coverage of the reads map back to TKHEV. The height of the graph at each base position represents the number of bases which means the number of sequencing times (log scale). The structure of genome sequence and predicted conserved domains of TKHEV were presented in the bottom panel. **B** Phylogenetic relationship of TKHEV based on RdRp domain (458aa) of members within *Hepeviridae*. Nodes with bootstrap values > 70 are noted. The red name indicates sequence obtained in this study

**Fig. 3:**
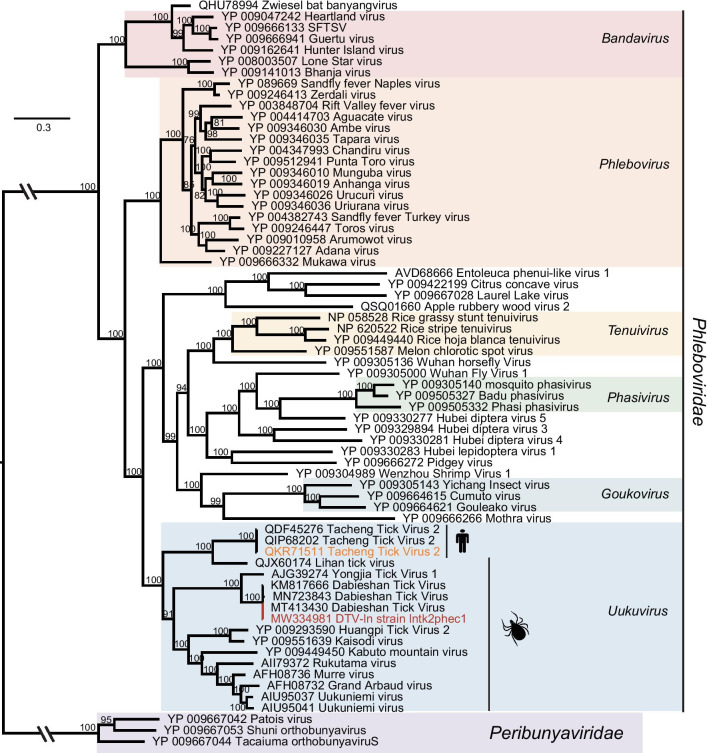
Phylogenetic trees of DTV-ln based on RdRp (920aa) sequence of members within *Phenuiviridae*. Nodes with bootstrap values > 70 are noted. The red name indicates sequence obtained in this study. The orange name indicates sequence obtained from human sample

**Fig. 4:**
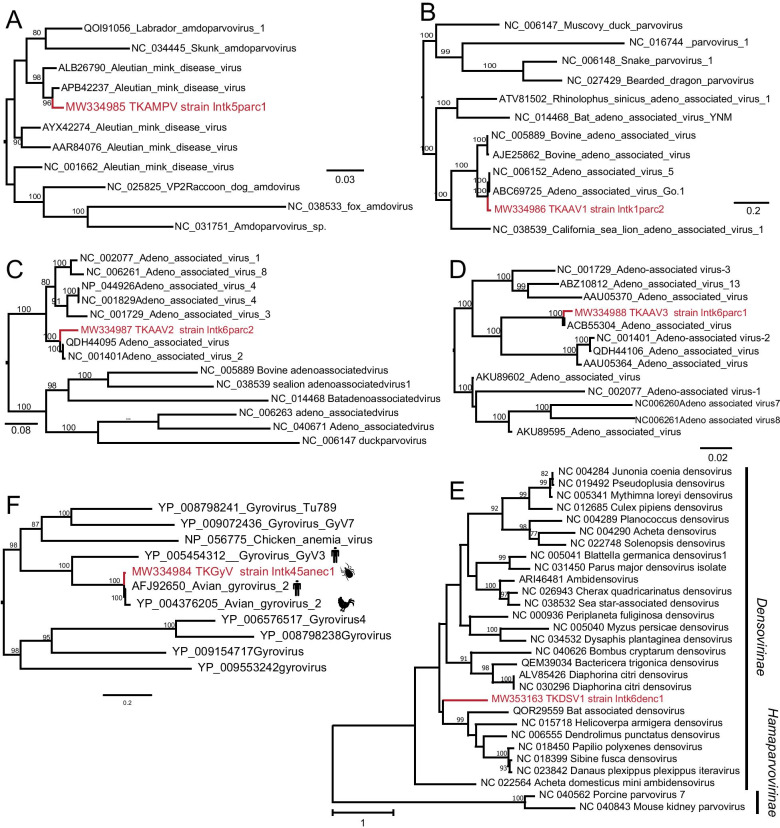
Phylogenetic trees of virus sequences within *Parvoviridae* and TKGyV. **A**, **D** Phylogenetic analysis based on capsid protein (145aa and 641aa, respectively). **B**–**C**, **E** Phylogenetic analysis based on replication protein (143aa, 172aa and 557aa, respectively). **F** Phylogenetic analysis based on VP1 (397aa) of gyrovirus. Nodes with bootstrap values > 70 are noted. The red name indicates sequences obtained in this study

**Fig. 5:**
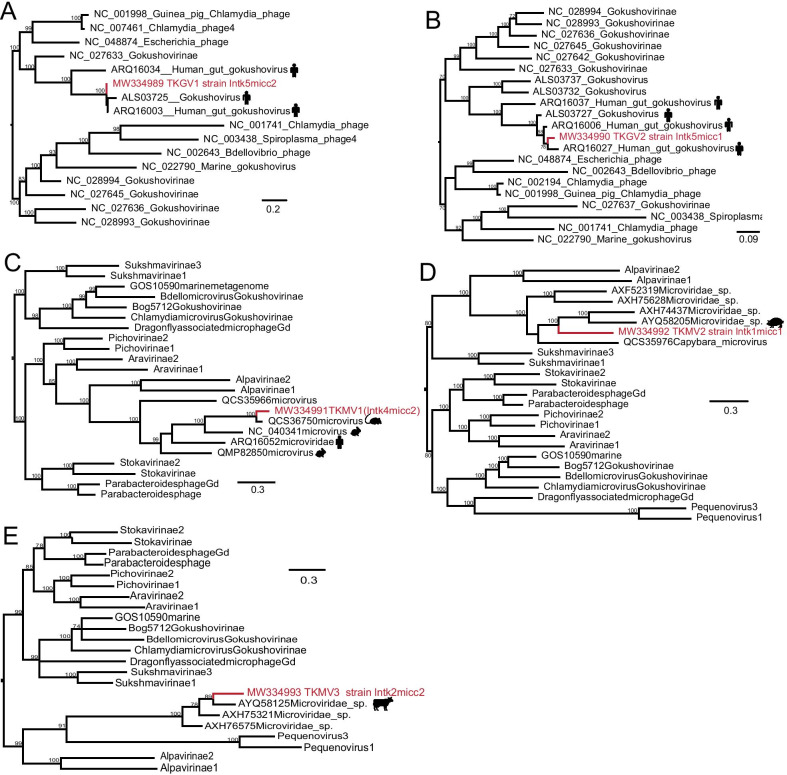
Phylogenetic trees of virus sequences within *Microviridae*. **A** Phylogenetic analysis based on replication protein (139aa). **B**–**E** Phylogenetic analysis based on capsid protein (491aa, 186aa, 160aa and 131aa, respectively)). Nodes with bootstrap values > 70 are noted. The red name indicates sequences obtained in this study

The sequence alignment files to build phylogenetic trees in Figs. 4 and 5 were made with 'ClustalW', and other alignment files to build phylogenetic trees in Figs. 2 and 3 were generated using 'MUSCLE'. The ClustalW [24] and MUSCLE [25] multiple sequence alignment programs in MEGA version 10.1.8 were run with default parameters to generate amino acid sequence alignments, including the sequences found in this study and the best BLASTx matches in GenBank and representative sequences from their corresponding family. One of the phylogenetic analyses (Fig. 4E) was only performed by maximum likelihood (ML) using MEGA version 10.1.8 [26] and the remaining phylogenetic tree by Bayesian inference (BI) methods using Mrbayes version 3.2.7 [27] based on amino acid sequences. The node supports were determined with 1000 bootstrap replicates in ML analyses. In the BI analyses, we used two simultaneous runs of Markov chain Monte Carlo sampling, and the runs were terminated upon convergence (standard deviation of the split frequencies < 0.01). The visualization and beautification of the phylogenetic tree were achieved by Figtree version 1.4.4.

## Results

### Overview of tick virome

A total of 300 adult ticks were collected across a forest park in southern Dalian City from June to August 2015. The samples represented one tick species common to Liaoning Province, *H. longicornis*. Ticks were pooled into six libraries based on the sampling period. All tick pools were sequenced by two lanes of Illumina HiSeq, resulting in 1,218,388 raw reads with an average of 203,064 raw reads per pool (Additional file 1). Of those reads, 5643 (0.463%) be identified as viral sequences through BLASTx search based on protein sequence identity, showing the percentage of viral reads in total reads was deficient (Fig. 1B). We found that viral reads clustered within known thirteen viral families. The presence and abundances of viral families from different pools are shown in Additional file 2, presenting the abundance of each family, which shows a vast difference (Fig. 1C). The viral reads contain 870 sequences, annotated as vertebrate viruses, 4519 annotated sequences as insect viruses, 131 annotated sequences as plant viruses, and 123 sequence reads as phage.

### A novel hepe-like virus sequence

Although there was imparity in virus sequence composition among libraries, reads of the single-stranded negative-sense viruses were the most abundant in the tick libraries, comprising 69% of all viral reads. After De Nove Assembly and Mapping to reference sequences, we recovered a 5.3 kb segment encoding a putative > 1,700-amino acid protein, provisionally named tick-borne hepe-like virus (TKHEV) strain lntk2unc1 (GenBank ID: MW334982; Protein ID: QTE18640) (Table 1). By mapping the original reads to this segment, the result indicates that the depth of sequencing is sufficient for the subsequent analysis (Fig. 2A). The closest relative of TKHEV, 52% amino acid sequence identity, was tick-borne tetravirus-like virus (KM048322) identified in *Dermacentor variabilis* ticks [28]. To detect this highly divergent virus sequence, we performed domain-based searches by Pfam with an expected value threshold of 1 × 10^−3^. The incomplete ORF contains three conserved domains, methyltransferase, RNA helicase, and RNA-dependent RNA polymerase (RdRp) domains (Fig. 2A), consistent with other virus sequences' genome structure of the family *Hepeviridae*. We also found limited amino acid identity (< 30%) across the RDRP domain of TKHEV with corresponding members of Hepeviridae through BLASTx search. Unfortunately, no read is assigned to the capsid protein region of TKHEV in all pools. The RdRp is the only conserved-sequence domain across all RNA virus sequences and was used for phylogenetic inference [30].

**Table 1:** Viral sequences identified in our study

Family	Name	Abbreviation	Length (bp)	Identity (BLASTx)	Coverage	Closest relative virus	Hits in rawdata	Genbank ID
*Hepeviridae*	Tick-borne hepe-like virus strain lntk2unc1	TKHEV	5130	52.65%	96.00%	Tick borne tetravirus-like virus	5552	MW334982
*Phleboviridae*	Tick uukuvirus strain lntk2phec1	DTV-ln	6263	99.81% (L)	99.00%	Dabieshan tick virus	955	MW334981
			180	96.67% (S)	100.00%	Dabieshan tick virus	2	MW334983
*Parvoviridae*	Tick amdoparvovirus strain lntk5parc1	TKAMPV	444	97.97%	100.00%	Aleutian mink disease virus	4	MW334985
	Tick adeno associated virus 1 strain lntk1parc2	TKAAV1	330	96.36%	100.00%	Adeno-associated virus	2	MW334986
	Tick adeno associated virus 2 strain lntk6parc2	TKAAV2	549	97.81%	100.00%	Adeno-associated virus	14	MW334987
	Tick adeno associated virus 3 strain lntk6parc1	TKAAV3	2665	96.28% (replication)	99.00%	Adeno-associated virus	69	MW334988
				99.25% (capsid)	100.00%	Adeno-associated virus		
	Tick densovirus 1 strain lntk6denc1	TKDSV1	1334	33.77%	81.00%	Cherax quadricarinatus densovirus	41	MW353163
	Tick densovirus 2 strain lntk6denc2	TKDSV2	725	27.50%	95.00%	lone star tick densovirus 1	21	MW353164
*Anelloviridae*	Tick gyrovirus strain lntk45anec1	TKGyV	1208	99.10%	82.00%	Avian gyrovirus 2	46	MW334984
*Microviridae*	Tick gokushovirus 1 strain lntk5micc2	TKGV1	471	99.36%	99.00%	Human gut gokushovirus	10	MW334989
	Tick gokushovirus 2 strain lntk5micc1	TKGV2	1519	96.67%	86.00%	Human gut gokushovirus	66	MW334990
	Tick microvirus 1 strain lntk4micc2	TKMV1	471	92.66%	100.00%	Microviridae sp.	20	MW334991
	Tick microvirus 2 strain lntk1micc1	TKMV2	453	87.42%	100.00%	Microviridae sp.	3	MW334992
	Tick microvirus 3 strain lntk2micc2	TKMV3	390	70.45%	100.00%	Microviridae sp.	2	MW334993

Strain name consists of four parts: Province acronyms + Pools no. + The first three letters Family or subfamily + Contig no

The phylogenetic tree shows that the hepatitis E viruses (HEVs) clustered into two major branches infecting vertebrates and invertebrates, based on three conserved domains, respectively. TKHEV was located in the branch of HEVs infecting invertebrates. TKHEV formed a distinct clade with Barns Ness breadcrumb sponge hepe-like viruses 1 and 2 (MF189998 and MF189999) (Fig. 2B), despite a very long branch separating them (KM048322 and MN830235 belong to unclassified viruses). Remarkably, TKHEV did not fall within the already classified genera and may represent a novel member of hepe-like virus in the family *Hepeviridae*. Moreover, it may represent a new family of arthropod-associated viruses.

### Uukuvirus sequence

Uukuvirus is a genus containing a clade of tick-borne phenuiviruses separated from the Phlebovirus and Banyangvirus (Bandavirus) clades [31]. Among the total reads, 9.7% of the reads were taxonomically related to a member of invertebrate single-stranded negative-sense viruses, from which we assembled a near-complete viral L segment and a short S segment. However, we did not obtain the segment of M, a critical component of the genome that encodes the viral glycoprotein allowing cell entry. The L segment and S segment tentatively were designated as Dabieshan tick virus (DTV-ln) strain lntk2phec1 (L: MW334981; S: MW334983) (Table 1). The L segment shows 99.81%, 99.52%, and 96.83% amino acid identity with Dabieshan tick virus (DTV) (MT413430, KM817666, and MN723843, respectively). These three were identified in *H. longicornis* ticks in China [10, 32]. The S segment shows a 96.67% amino acid identity with DTV (KM817733), which was also collected from *H. longicornis* ticks [10]. Based on RdRp, DTV-ln formed a well-supported monophyletic group closely related to the "classic" uukuviruses, suggesting that they may share a single common ancestor in these genes. Interestingly, a group of Tacheng tick virus 2 (TcTV2) and Lihan tick virus formed a separate branch that was previous to a branch of DTV-ln. Of these, TcTV2 (QKR71511), a novel phlebovirus, was identified from a patient with a history of tick bite in northwestern China and caused the patient headache, anorexia, nausea, vomiting, fever, neck stiffness, and erythema [33]. DTV-ln fell with genus *Uukuvirus* and formed a branch with a group of DTVs [10, 34] (Fig. 3A), suggesting DTV-ln likely can infect people.

### Parvoviridae sequences

In this study, six viral sequences clustered within the family *Parvoviridae*. Among these, four sequences belong to the subfamily *Parvovirinae* infecting vertebrates (including humans), and the other two sequences belong to the subfamily *Densovirinae* infecting invertebrates [35]. A segment from amdoparvovirus, tentatively designated as tick amdoparvovirus (TKAMPV) strain lntk5parc1 (MW334985), was identified. TKAMPV had a high identity (98.62%) at the amino acid level to Aleutian mink disease virus. Phylogenetic trees showed TKAMPV clustered with Aleutian mink disease virus (APB42237 and ALB26790), isolated from mink sampled from China (Fig. 4A). Three sequence fragments fell with the genus *Dependoparvovirus* were identified. Due to high identity with adeno associated virus, we tentatively named it tick adeno associated virus 1 (TKAAV1) strain lntk1parc2 (MW334986), tick adeno associated virus 2 (TKAAV2) strain lntk6parc2 (MW334987), and tick adeno associated virus 3 (TKAAV3) strain lntk6parc1 (MW334988) (Table 1). TKAAV1 and TKAAV2 encode a replication-associated protein, and another segment encodes partial replication-associated and capsid protein. The amino acid sequences of TKAAV1, TKAAV2, and TKAAV3 all share high identities (> 96%) with adeno-associated viruses. Phylogenetic trees suggested the above three viral sequences clustered into the group of adeno associated viruses within genus *Dependoparvovirus* (Fig. 4B–D).

The other two segments shared < 40% amino acid identity with the more closely related virus, tentatively named tick densovirus 1 (TKDSV1) strain lntk6denc1(MW353163) and tick densovirus 2 (TKDSV2) strain lntk6denc2 (MW353164). TKDSV1 formed an individual cluster before a group of viruses belonging to Iteradensovirus (Fig. 4E), showing it may be a new genus of *Densovirinae*. In total, we only make sure TKDSV1 belongs to the subfamily *Densovirinae*, but we still cannot determine its specific genus.

### Gyrovirus sequence

We identified a segment that belonged to the family *Anelloviridae* and named it tick gyrovirus (TKGyV) strain lntk45anec1 (MW334984) (Table 1). TKGyV had an incomplete open reading frame (ORF), exhibiting 99% amino acid identity with avian gyrovirus 2 (AFJ92650), a virus isolated from human fecal samples. Based on the VP1, phylogenetic analysis indicated that TKGyV is inside a clade containing the avian gyrovirus 2 (AFJ92650, YP004376205) (Fig. 4F).

### Microviridae sequences

We identified five segments within the family *Microviridae*, named tick gokushovirus 1 (TKGV1) strain lntk5micc2 (MW334989), tick gokushovirus 2 (TKGV2) strain lntk5micc1(MW334990), tick microvirus 1 (TKMV1) strain lntk4micc2 (MW334991), tick microvirus 2 (TKMV2) strain lntk1micc1 (MW334992) and tick microvirus 3 (TKMV3) strain lntk2micc2 (MW334993) (Table 1). Phylogenetic trees using the replication protein (VP4) amino acid revealed that TKGV1 clusters with an unclassified *Gokushovirinae*, which was isolated from *Homo sapiens* (ARQ16003), with 99.4% amino acid sequence identity (Fig. 5A), TKGV2 formed a clade with Gokushoviruses, with 96.7% amino acid sequence identity (Fig. 5B). The other segments are highly divergent from all other microviruses currently available, exhibiting only 38%-84% amino acid identity. TKMV2 formed a clade with unclassified Microvirus (AXH74437, AYQ58205) (Fig. 5D), while TKMV1 and TKMV3 formed a separate clade for more distantly related to other microviruses, which suggests these maybe represent the segments of the novel virus.

## Discussion

Liaoning Province is rich in forests and wildlife, providing an adaptable condition to live for ticks. Researchers found that more enormous forests had more extensive and more diverse wildlife communities, which supported more extensive and diverse tick communities [36]. In nature, environments in the forest, including ticks and other animals, are usually in equilibrium. However, the enlargement of cities and exposure of wild hosts to humans and livestock animals have destroyed the balance between them, resulting in the risk of infection increased annually, especially farmers living in wooded and hilly areas [37] and tourists. Furthermore, *H. longicornis* is found to harbor the highest variety of tick-borne agents [38]. At least 30 human pathogens were linked with *H. longicornis*, including six species of virus: Severe fever with thrombocytopenia syndrome virus, Jingmen tick virus, Bocavirus, Nairobi sheep disease virus, Lymphocytic choriomeningitis virus, Tick-borne encephalitis virus [39].

Our primary objective is to throw light on the virome of 300 adult *H. longicornis* ticks in Liaoning Province. Two lanes of Illumina HiSeq were used to generate 1,218,388 raw reads from six tick pools; of these, 5665 (0.465%) reads belong to 18 viral families. Notably, we observed a phenomenon regarding the reads number from Pool02 is exceptionally high (Fig. 1B, C). On the one hand, this may be due to the difference between the tick samples because pools to each other did not differ in steps of sample treatment as nucleic acid extraction, cDNA synthesis, and library construction. On the other hand, the sample volume of each library is based on the relative brightness intensity of electrophoretic bands to determine to safeguard consistency in each library in an integrated library to purification and sequencing. However, the limitation of this approach is that sample volume may not be of sufficient accuracy that causes some errors between every pool.

Previous meta-transcriptomic studies targeting arthropod viromes have revealed abundant novel and highly divergent viruses [30, 40]. Totally fourteen virus sequences concerning hepe-like, phlebo-, parvo-, anello- and microviruses were identified, indicating the circulation of diverse tick-associated viruses in Liaoning. Based on updates of the viral database, we compared our results with those sequences from previous studies [22, 41]. TKHEV, an hepe-like virus, possessed an incomplete ORF with three conserved domains, consistent with other viruses' genome structure within the family *Hepeviridae*. TKHEV had 52% amino acid sequence identity with tick-borne tetravirus-like virus (AII01815) and limited amino acid identity across the RDRP domain of this virus with a representative of Hepeviridae. The tick-borne tetravirus-like virus tentatively divided into the Alphatetraviridae, due to dissimilarity in sequence and host association, it likely represents a new family of arthropod-associated viruses [28, 42]. According to ICTV, members of the family *Hepeviridae* are classified into two genera *Orthohepevirus* infecting mammals and avians, and *Piscihepevirus*, infecting invertebrates [29, 43]. Based on the RDRP domain, TKHEV fell with a branch infecting invertebrates (Fig. 2B). By characterizing the genomic organization, phylogenetic relationship, and conserved motifs, we found that this new virus may pertain to a new genus in the family *Hepeviridae* because it has a similar conserved genome structure highly divergent from the known members in the family (Fig. 2B). To our knowledge, it is the earliest hepe-like virus identified from ticks. Furthermore, TKHEV was highly prevalent in ticks sampled with a rate of 50%, and the pathogenicity needs further study.

The next section of our study was concerned with the transmission of DTV. A strain of DTV (KM817666) was first discovered in *H. longicornis* ticks in Hubei province of China in 2014 [10]. Researchers found fragments S and L of another strain of DTV (MT413430) in Shandong province in 2020. DTV-ln also were detected in *H. longicornis* ticks in Liaoning Province, suggesting the DTV is highly prevalent and widely distributed in China. However, ticks have very restricted distribution and need the host's blood to live, which is why we speculate that they require an artificial or another way to move. Not only that, Liaoning has rich animal resources, especially birds. Therefore, we hypothesize that the migration of birds provides a geographic link between viruses [44]. Epidemiological investigations showed that the reads of the L segment of DTV-ln were detected positive in four out of six pools (66.7%), but the S segment was only detected in pool 02. Besides, DTVs show a high amino acid identity with each other, suggesting they are relatively conservative in evolution and host selection. In our opinion, DTV is likely to be widespread in other regions of China as a natural focus pathogen. Due to the lack of related research on the DTV, its pathogenicity is still unknown. It may be a potential threat to human beings and animals, which deserves constant attention. Phylogenetic analysis showed that DTVs belonged to the genus *Uukuvirus*. Moreover, a group of Tacheng tick virus 2 (TcTV2) and Lihan tick virus formed a separate branch that was previous to a branch of DTV-ln. One of these (QKR71511) can cause severe symptoms, suggesting DTV-ln likely can infecting people and other animals.

The researchers found avian gyrovirus 2 (AGV2) in the serum of diseased chicken for the first time [45], but to date, no evidence of AGV2 causing disease has been produced [46]. Our study found TKGyV including VP1 and VP2 matched to AGV2, suggesting ticks may transmit this virus to chickens by bites. Besides, a novel fragment in Densovirinae was detected, which helped us know more about viruses infecting ticks.

The single most noteworthy observation to emerge from our study was that the sequences matched to mammalian viruses were found in the six libraries, such as TKAMPV, and TKAAV1-3 (Table 1). Low abundance of sequence reads matched to mammalian viruses in all the tick libraries suggests that they probably do not really replicate in ticks but from the blood meal of the ticks. Whether these mammalian viruses carried by ticks can cause tick-borne transmission to human or other mammals needs further research.

## Conclusions

Summarily, we provide a comprehensive investigation on virome in free ticks in Liaoning. Though no known viruses causing diseases to humans and animals were detected, our results can confirm the presence of uuku-, hepe-like, parvo-, gyro- and microvirus sequences in ticks. Nevertheless, one restraint of our study was the limited geographical distribution of the sampled ticks. We expect that analysis of ticks from diverse geographical areas would uncover more remarkable viral diversity of tick-borne viruses. Our study emphasizes a prominent diversity of the virus community and extends the present viral diversity. However, further research is needed to uncover the role of viruses in the pathogenic mechanism in animals and humans.

## Supplementary Information

**Additional file 1.** Summary of sampling time, sits, type, species and sequenced data of six pools. (.csv)

**Additional file 2.** Presence and abundances of viral families from different pools. (.csv)

## Data Availability

The datasets supporting the conclusions of this article are available in NCBI. The raw data (Pool 01-06) of NGS sequencing were deposited in NCBI GenBank under accession numbers SRR13164785, SRR13164887, SRR13164889, SRR13164897, SRR13164899, and SRR13164901, respectively. The GenBank accession numbers for the sequences are MW334981-MW334993 and MW353163-MW353164.

## Abbreviations

NCBI: National Center for Biotechnology Information
ICTV: International Committee on Taxonomy of Viruses
NVNR: Non-virus non-redundant protein database
BLAST: Basic Local Alignment Search Tool

## References

[1] Jongejan F, Uilenberg G (2004). The global importance of ticks. Parasitology.

[2] Kernif T, Leulmi H, Raoult D, Parola P (2016). Emerging tick-borne bacterial pathogens. Microbiol Spectr.

[3] Tsergouli K, Karampatakis T, Haidich AB, Metallidis S, Papa A (2020). Nosocomial infections caused by Crimean-Congo haemorrhagic fever virus. J Hosp Infect.

[4] Zhan J, Wang Q, Cheng J, Hu B, Li J, Zhan F, Song Y, Guo D (2017). Current status of severe fever with thrombocytopenia syndrome in China. Virol Sin.

[5] Jaenson TG, Jaenson DG, Eisen L, Petersson E, Lindgren E (2012). Changes in the geographical distribution and abundance of the tick Ixodes ricinus during the past 30 years in Sweden. Parasit Vectors.

[6] Tijsse-Klasen E, Koopmans MP, Sprong H (2014). Tick-borne pathogen - reversed and conventional discovery of disease. Front Public Health.

[7] Rodino KG, Theel ES, Pritt BS (2020). Tick-borne diseases in the United States. Clin Chem.

[8] Zhang YZ, Chen YM, Wang W, Qin XC, Holmes EC (2019). Expanding the RNA virosphere by unbiased metagenomics. Annu Rev Virol.

[9] Zhang Y-Z, Shi M, Holmes EC (2018). Using metagenomics to characterize an expanding virosphere. Cell.

[10] Li CX, Shi M, Tian JH, Lin XD, Kang YJ, Chen LJ, Qin XC, Xu J, Holmes EC, Zhang YZ (2015). Unprecedented genomic diversity of RNA viruses in arthropods reveals the ancestry of negative-sense RNA viruses. Elife.

[11] Qin XC, Shi M, Tian JH, Lin XD, Gao DY, He JR, Wang JB, Li CX, Kang YJ, Yu B (2014). A tick-borne segmented RNA virus contains genome segments derived from unsegmented viral ancestors. Proc Natl Acad Sci USA.

[12] Zhang YZ, Zhou DJ, Qin XC, Tian JH, Xiong Y, Wang JB, Chen XP, Gao DY, He YW, Jin D (2012). The ecology, genetic diversity, and phylogeny of Huaiyangshan virus in China. J Virol.

[13] Mansfield KL, Jizhou L, Phipps LP, Johnson N (2017). Emerging tick-borne viruses in the twenty-first century. Front Cell Infect Microbiol.

[14] Bush S, Reed M, Maher S (2013). Impact of forest size on parasite biodiversity: implications for conservation of hosts and parasites. Biodivers Conserv.

[15] Jia N, Wang J, Shi W, Du L, Sun Y, Zhan W, Jiang J-F, Wang Q, Zhang B, Ji P (2020). Large-scale comparative analyses of tick genomes elucidate their genetic diversity and vector capacities. Cell.

[16] Zhao T, Gong H, Shen X, Zhang W, Shan T, Yu X, Wang SJ, Cui L (2020). Comparison of viromes in ticks from different domestic animals in China. Virol Sin.

[17] Walker AR, Matthews J, Preston PM (2005). The development of electronic keys for the identification of ticks. Int J Trop Insect Sci.

[18] Zhang W, Li L, Deng X, Blumel J, Nubling CM, Hunfeld A, Baylis SA, Delwart E (2016). Viral nucleic acids in human plasma pools. Transfusion.

[19] Zhang W, Li L, Deng X, Kapusinszky B, Pesavento PA, Delwart E (2014). Faecal virome of cats in an animal shelter. J Gen Virol.

[20] Zhang W, Yang S, Shan T, Hou R, Liu Z, Li W, Guo L, Wang Y, Chen P, Wang X (2017). Virome comparisons in wild-diseased and healthy captive giant pandas. Microbiome.

[21] Altschul SF, Tl M, Schäffer AA, Zhang J, Zhang Z, Miller W, Lipman DJ (1997). Gapped BLAST and PSI-BLAST: a new generation of protein database search programs. Nucleic Acids Res.

[22] Deng X, Naccache SN, Ng T, Federman S, Li L, Chiu CY, Delwart EL (2015). An ensemble strategy that significantly improves de novo assembly of microbial genomes from metagenomic next-generation sequencing data. Nucleic Acids Res.

[23] Kearse M, Moir R, Wilson A, Stones-Havas S, Cheung M, Sturrock S, Buxton S, Cooper A, Markowitz S, Duran C (2012). Geneious basic: an integrated and extendable desktop software platform for the organization and analysis of sequence data. Bioinformatics (Oxford, England).

[24] Thompson JD, Higgins DG, Gibson TJ (1994). CLUSTAL W: improving the sensitivity of progressive multiple sequence alignment through sequence weighting, position-specific gap penalties and weight matrix choice. Nucleic Acids Res.

[25] Edgar RC (2004). MUSCLE: multiple sequence alignment with high accuracy and high throughput. Nucleic Acids Res.

[26] Kumar S, Stecher G, Li M, Knyaz C, Tamura K (2018). MEGA X: molecular evolutionary genetics analysis across computing platforms. Mol Biol Evol.

[27] Huelsenbeck JP, Ronquist F (2001). MRBAYES: Bayesian inference of phylogenetic trees. Bioinformatics.

[28] Tokarz R, Williams SH, Sameroff S, Sanchez Leon M, Jain K, Lipkin WI (2014). Virome analysis of *Amblyomma americanum*, *Dermacentor variabilis*, and *Ixodes scapularis* ticks reveals novel highly divergent vertebrate and invertebrate viruses. J Virol.

[29] Purdy MA, Harrison TJ, Jameel S, Meng XJ, Okamoto H, Van der Poel WHM, Smith DB, Ictv Report C (2017). ICTV virus taxonomy profile: Hepeviridae. J Gen Virol.

[30] Shi M, Lin XD, Tian JH, Chen LJ, Chen X, Li CX, Qin XC, Li J, Cao JP, Eden JS (2016). Redefining the invertebrate RNA virosphere. Nature.

[31] King AMQ, Lefkowitz E, Adams MJ, Carstens EB (2011). Virus taxonomy: ninth report of the international committee on taxonomy of viruses.

[32] Shao L, Pang Z, Fu H, Chang R, Lin Z, Lv A, Wang S, Kong X, Luo M, Liu X (2020). Identification of recently identified tick-borne viruses (Dabieshan tick virus and SFTSV) by metagenomic analysis in ticks from Shandong Province, China. J Infect.

[33] Dong Z, Yang M, Wang Z, Zhao S, Xie S, Yang Y, Liu G, Zhao S, Xie J, Liu Q, Wang Y (2021). Human Tacheng tick virus 2 infection, China, 2019. Emerg Infect Dis.

[34] Kobayashi D, Murota K, Itokawa K, Ejiri H, Amoa-Bosompem M, Faizah AN, Watanabe M, Maekawa Y, Hayashi T, Noda S (2020). RNA virome analysis of questing ticks from Hokuriku District, Japan, and the evolutionary dynamics of tick-borne phleboviruses. Ticks Tick-Borne Dis.

[35] Cotmore SF, Agbandje-McKenna M, Canuti M, Chiorini JA, Eis-Hubinger A-M, Hughes J, Mietzsch M, Modha S, Ogliastro M, Pénzes JJ (2019). ICTV virus taxonomy profile: Parvoviridae. J Gen Virol.

[36] Esser HJ, Herre EA, Kays R, Liefting Y, Jansen PA (2019). Local host-tick coextinction in neotropical forest fragments. Int J Parasitol.

[37] Yu XJ, Liang MF, Zhang SY, Liu Y, Li JD, Sun YL, Zhang L, Zhang QF, Popov VL, Li C (2011). Fever with thrombocytopenia associated with a novel bunyavirus in China. N Engl J Med.

[38] Zhao GP, Wang YX, Fan ZW, Ji Y, Liu MJ, Zhang WH, Li XL, Zhou SX, Li H, Liang S (2021). Mapping ticks and tick-borne pathogens in China. Nat Commun.

[39] Zhao L, Li J, Cui X, Jia N, Wei J, Xia L, Wang H, Zhou Y, Wang Q, Liu X (2020). Distribution of *Haemaphysalis longicornis* and associated pathogens: analysis of pooled data from a China field survey and global published data. Lancet Planet Health.

[40] Shi M, Lin XD, Vasilakis N, Tian JH, Li CX, Chen LJ, Eastwood G, Diao XN, Chen MH, Chen X (2016). Divergent viruses discovered in arthropods and vertebrates revise the evolutionary history of the Flaviviridae and related viruses. J Virol.

[41] Bolling BG, Vasilakis N, Guzman H, Widen SG, Wood TG, Popov VL, Thangamani S, Tesh RB (2015). Insect-specific viruses detected in laboratory mosquito colonies and their potential implications for experiments evaluating arbovirus vector competence. Am J Trop Med Hyg.

[42] Tokarz R. Identification of novel viruses in *Amblyomma americanum*, *Dermacentor variabilis*, and *Ixodes scapularis* ticks. Am Soc Microbiol. 2018. 10.1128/mSphere.00614-17 PMC5853492 29564401

[43] Wu N, Zhang P, Liu W, Wang X (2018). Sogatella furcifera hepe-like virus: first member of a novel Hepeviridae clade identified in an insect. Virus Res.

[44] Wille M, Harvey E, Shi M, Gonzalez-Acuna D, Holmes EC, Hurt AC (2020). Sustained RNA virome diversity in Antarctic penguins and their ticks. ISME J.

[45] Rijsewijk FA, Dos Santos HF, Teixeira TF, Cibulski SP, Varela AP, Dezen D, Franco AC, Roehe PM (2011). Discovery of a genome of a distant relative of chicken anemia virus reveals a new member of the genus Gyrovirus. Arch Virol.

[46] Varela AP, Dos Santos HF, Cibulski SP, Scheffer CM, Schmidt C, Sales Lima FE, Silva AD, Esteves PA, Franco AC, Roehe PM (2014). Chicken anemia virus and avian gyrovirus 2 as contaminants in poultry vaccines. Biologicals.

